# Commentary: Microstructure, length, and connection of limbic tracts in normal human brain development

**DOI:** 10.3389/fnins.2017.00117

**Published:** 2017-03-13

**Authors:** Kenichi Oishi

**Affiliations:** The Russell H. Morgan Department of Radiology and Radiological Science, The Johns Hopkins University School of MedicineBaltimore, MD, USA

**Keywords:** default mode network, development, diffusion tensor imaging, limbic tracts, free water elimination, neonate, resting-state fMRI, tractography

Limbic tracts are affected in various neurological and psychiatric diseases/disorders (Bogerts et al., 1985; Modell et al., 1989; Braak and Braak, 1991; Tamminga et al., 1992; Becker et al., 2001; Amaral et al., 2008; Sheth et al., 2013; Gottlich et al., 2014; Posner et al., 2014). The onset of the abnormality is of particular interest since many psychiatric disorders are known to have genetic backgrounds that might result in a specific phenotype of the brain anatomy (endophenotype) before the onset of the symptoms (Menzies et al., 2008; Hajek et al., 2009; Fornito et al., 2013; Nery et al., 2013; Dixson et al., 2014; Scognamiglio and Houenou, 2014; Zannas et al., 2014; Chakravarty et al., 2015; Ordóñez et al., 2016). There is the potential that such endophenotypes could be detected even in very early developmental stages, therefore, quantification of the developmental status of the limbic fibers might be useful for identifying groups at high-risk for developing psychiatric disorders in the future. However, little is known about the normal developmental trajectories of these fiber tracts from a neonatal age to young adulthood, which is essential for the evaluation of a pathological deviation from normal brain development (Oishi et al., 2013). MRI scans are particularly challenging in subjects less than 4 years of age without sedation (Oishi et al., 2012), and therefore, establishing the normal developmental trajectory of this age-range will be an important asset for research communities(Oishi et al., 2011; Akazawa et al., 2015; Chang et al., 2016).

Yu and his colleagues aimed to identify the normal developmental characteristics of the limbic fibers (Yu et al., 2014). They used diffusion tensor imaging (DTI) to characterize the microscopic, anatomical features of the live human brain from the neonatal period to 25 years of age, based on cross-sectional observation of 65 healthy individuals. Diffusion property, length, and anatomical connections of the three limbic tracts were evaluated: The cingulate gyrus part of the cingulum (cgc); the hippocampal part of the cingulum (cgh); and the fornix. The tracts-of-interest (TOI) approach was applied to accurately identify the white matter tracts and has been used to evaluate the microscopic status of the developing brain. Tractography was also used to measure the length of the limbic tracts, as well as to evaluate the connectivity of the anatomical structures that are related to the default mode network (DMN), which is defined by resting-state fMRI. In adult brains, the limbic fibers are known to connect the DMN-related structures. However, the role of these limbic fibers in connecting DMN structures has been unclear in early development.

There were three major findings. First, the developmental curve of four DTI-derived measures were mostly logarithmic, with rapid changes until 2 years of age, followed by slow changes that went on until 25 years of age. The important contribution to science here is that these results were compared with and without free water elimination (FWE) (Pasternak et al., 2009), which was introduced to eliminate the partial volume artifact that is caused by the inclusion of cerebrospinal fluid (CSF) into each pixel, which results in falsely high diffusivity of such pixels. There was a concern about whether to apply the FEW to brains under development, since it could potentially eliminate the effect of physiological changes of the brain related to development by eliminating the intercellular water content that is prominent in early development. The authors first demonstrated that contamination of the CSF is the major source of free water that is eliminated by the FWE algorithm. Importantly, the application of the FWE did not change the pattern of the developmental curves of the limbic fibers, which means that both values, with and without FWE, are legitimate for the evaluation of brain development, as long as the method for evaluation is consistent. Second, the authors demonstrated that the length of the cgc, normalized by the anterior-to-posterior length of the brain, increased with age, but the normalized length of the cgh and the fornix were unchanged. This means that the development of the cgc is disproportionally rapid during this age-range, but the development of the cgh and the fornix is proportional. Third, the anatomical connections of DMN-related structures were similar in both neonates and young adults, which means that the functional and anatomical connectivity of the DMN is already established in the early postnatal period. This suggested the importance of the DMN in the basic brain functions that are already needed at birth.

The simultaneous evaluation of the DTI-derived measures—length and connectivity of the limbic fibers from early to late development—provide an important foundation with which to assess the types and onset of abnormalities in brain development related to various neurological or psychiatric disorders. Indeed, during the two years since publication, the results of this paper were referenced to interpret DTI findings of traumatic brain injury (TBI) and major depressive disorder (MDD); the left-side dominancy in the effect of TBI was interpreted in relation to the leftward asymmetry observed in normal development (Ewing-Cobbs et al., 2016), and disrupted functional connectivity in MDD was interpreted in the context of chronological patterns in white matter maturation (Sacchet et al., 2016).

Several limitations should be noted. The limited number of participants might introduce a selection bias. The limbic pathways investigated were limited; the uncinate fasciculus, stria terminalis, and the mammillothalamic fasciculus were not included. The parameters investigated from diffusion MRI were limited to those derived from the tensor model; parameters acquired through advanced models, such as diffusion spectrum imaging or high-angular resolution diffusion imaging, remain to be investigated. For the evaluation of intra-individual variation, a longitudinal design would be needed (Baltes, 1968). To overcome these general and well-recognized limitations, several longitudinal studies have been launched since the publication of this paper, including the Developing Human Connectome Project, Baby Connectome Project, Lifespan Human Connectome Project, and the Adolescent Brain Cognitive Development (ABCD) study. These projects adopted state-of-the-art scanners and scan protocols. Among them, the ABCD study is the largest study, and involves approximately 10,000 children, who will be followed for 9–10 years. These studies are expected to overcome the limitations of previous cross-sectional studies.

## Author contributions

KO contributed to all aspects of the work, including conception and writing, and is accountable for all aspects of this commentary.

## Funding

The author is supported by NIH grant R01HD065955 and an *in*Health Pilot Project grant from the Johns Hopkins Individualized Health Initiative. The content of this commentary is solely the responsibility of the author and does not necessarily represent the official view of NIH/NICHD or the Johns Hopkins Individualized Health Initiative.

### Conflict of interest statement

The author declares that the research was conducted in the absence of any commercial or financial relationships that could be construed as a potential conflict of interest.
